# LEVERAGING ANALYTIC METHODS TO EXPAND OPPORTUNITIES IN AGING-RELATED HEALTH DISPARITIES RESEARCH

**DOI:** 10.1093/geroni/igz038.1592

**Published:** 2019-11-08

**Authors:** Igor Akushevich, Carl V Hill, Heather E Whitson

**Affiliations:** 1 Duke University, Durham, North Carolina, United States; 2 NIH/National Institute on Aging, Bethesda, Maryland, United States; 3 Duke University Medical Center, Durham, North Carolina, United States

## Abstract

The objective of the Symposium is to improve the understanding of how existing analytic methods and data can be leveraged to make progress in understanding the causes and mechanisms of health-related disparities in Alzheimer’s disease, related dementias and other prominent age-related diseases. Topics will cover a range of academic and administrative topics including: i) advanced analytic methods and modeling of health disparities with application to racial and geographic disparities in AD/ADRD; ii) the role of repeated anesthetic and surgical exposure in generation of disparities in AD/ADRD risk; iii) the nature of health disparities in cognitive aging as parallel to or distinct from health disparities in patterns of aging in other systems in the body; iv) recent advances in machine learning applied to large claims databases involving medical disparities; and v) geographic-related disparities in life expectancy across the U.S. A focus will be made on demonstrating how studies using established administrative data resources such as Medicare claims databases combined with innovative analytic approaches such as partitioning analyses, time-series based methods of projection and forecasting, and stochastic process models can be used to uncover previously overlooked or understudied aspects in this area of research. Analyses of such increasingly available large health datasets provides an opportunity to obtain nationally representative multiethnic results based on individual-level measures that reflect the real care-related and epidemiological processes ongoing in the U.S. healthcare system and allows the targetting of relatively rare diseases in relatively small population subgroups.

